# Physical Activity Promotion: A Systematic Review of The Perceptions of Healthcare Professionals

**DOI:** 10.3390/ijerph17124358

**Published:** 2020-06-18

**Authors:** Francis A. Albert, Melissa J. Crowe, Aduli E. O. Malau-Aduli, Bunmi S. Malau-Aduli

**Affiliations:** 1 College of Medicine and Dentistry, James Cook University, Townsville, QLD 4811, Australia; bunmi.malauaduli@jcu.edu.au; 2 Division of Tropical Health and Medicine, James Cook University, Townsville, QLD 4811, Australia; melissa.crowe@jcu.edu.au; 3 College of Public Health, Medical and Veterinary Sciences, James Cook University, Townsville, QLD 4811, Australia; aduli.malauaduli@jcu.edu.au

**Keywords:** physical activity promotion, healthcare professionals, primary healthcare, physical activity, physical inactivity

## Abstract

Physical activity (PA) is a cost-effective and non-pharmacological foundation for the prevention and management of chronic and complex diseases. Healthcare professionals could be viable conduits for PA promotion. However, the evidence regarding the effectiveness and benefits of the current forms of PA promotion are inconclusive. Healthcare professionals’ perceptions on key determinants impact on the optimum promotion of PA were explored in this review. Thirty-four (34) studies were identified after systematically searching seven databases for peer-reviewed articles published within the last decade. PA advice or counselling was the most recorded form of PA promotion, limited counselling time was the most reported obstacle while providing incentives was viewed as a key facilitator. There is widespread consensus among healthcare professionals (HCPs) on some aspects of PA promotion. Utilisation of all PA promotional pathways to their full potential could be an essential turning point towards the optimal success of PA promotional goals. Hence, strategies are required to broaden chronic disease treatment methods to include preventive and integrative PA promotion approaches particularly, between frontline HCPs (e.g., GPs) and PA specialists (e.g., EPs). Future studies could explore the functionality of GP to EP referral pathways to determining what currently works and areas requiring further development.

## 1. Introduction

Physical activity (PA) has been described as a wonder drug [1]; owing to its positive impacts on physical and mental wellbeing [2,3] and its ability to prevent disability [4] and myriads of chronic diseases [5]. World Health Organization (WHO) defines PA as any bodily movement produced by the skeletal muscles that requires increased energy expenditure above resting requirements and involves household tasks, leisure time activity, and structured physical activity [6]. Despite growing emphasis on the promotion of PA [7,8], the burden of physical inactivity (PI) continues to increase as over 35% of the global population fail to meet the recommended PA guidelines [9] and 5.3 million premature deaths are now directly linked to PI [10]. A 25% reduction in PI could prevent over 1.3 million deaths each year [11].

PA promotional programmes have been developed worldwide since the 1990s and are still utilised in various settings [12,13,14,15,16]. These programmes are typically structured and include PA counselling, advice on behavioural change and/or referral to specialists for an individualised PA programme within a healthcare context [17]. Public health policies are being used to motivate healthcare professionals (HCPs) towards the delivery of behavioural change activities like the promotion of PA to patients [18]. Additionally, measures advocating for the inclusion of PA into patients’ treatment plans have been initiated by several policies, and some notable examples include Healthy People 2020 [19] and Exercise is Medicine [20,21].

Various studies have reported PA promotion as an effective intervention in diverse healthcare settings [22,23,24] and that HCPs can be very useful conduits for promoting PA [24]. Successful intervention is attributed to the different levels of one-to-one contact a patient might have with different HCPs during treatment and the significant PA behavioural change that could ensue if PA was promoted at each contact thereby, making every contact count [25,26]. WHO and other agencies have reiterated that HCPs are pivotal to promoting PA and healthcare systems could be key avenues for reducing chronic diseases and PI [8,27,28]. Nonetheless, it has been argued that combined support of the academic and scientific communities would be required in synergy with the efforts of HCPs and policy makers to ultimately achieve the 2013–2020 WHO’s global action plan designed to achieve a 10% reduction in PI by 2025 [29].

However, the evidence regarding the key determinant factors that impact on the promotion of PA among HCPs are inconclusive [30,31]. Studies have claimed that several barriers hinder the effective promotion of PA in primary healthcare settings [32,33,34], and that several HCPs miss the opportunities to promote PA to their patients [31,35,36,37,38,39]. Further claims indicated that these opportunities could have been missed because of the brief and non-specificity of HCPs’ advice [40], lack of knowledge and confidence on the effective strategies for promoting PA [41,42], lack of skills, limited time, reimbursements, current workload, and practice barriers [43,44]. Other barriers include lack of training [45,46] and HCPs’ beliefs about a patient’s readiness to change PA habits [47,48]. If these barriers and the growing prevalence of chronic diseases and PI are not urgently addressed, there could be worsening cases of premature deaths, long-term disabilities, hospitalisation, rehabilitation costs [49], and burden on the healthcare systems [50].

Studies on the key determinants of the effectiveness and long-term sustainability of PA promotional goals from the perspective of HCPs are required [32]. The pressing need for the opinions of key PA stakeholders about key determinants of PA promotion and a sustainable integrated health solution to the growing burden of PI and chronic diseases, highlights the need for a systematic assessment and synthesis of current research on this topic [51,52,53]. This will help identify gaps in the literature and give direction for future research. A thorough review of literature would provide the information that could enhance PA promotional practices, optimise utilization of public health resources, and ultimately improve health outcomes for patients. Consequently, the main objective of this review was to examine relevant primary peer-reviewed articles in order to synthesize the research evidence on PA promotion from the perspective of HCPs. The secondary objective was to explore the key determinants impacting on the optimum achievement of PA promotional goals in healthcare systems.

Considering these objectives and the need to explicitly appraise and synthesize current evidence on the key determinants of effective PA promotion, a systematic review was deemed the most suitable approach for reviewing the literature [51,52,53]. Systematic reviews are studies often conducted for the purpose of identifying, appraising and integrating the evidence pertinent to specific question(s) in order to inform practice, policy, and further research [54,55].

The following questions were addressed by this review:

1. What are HCPs’ perceptions regarding key determinants of PA promotion?
2. What are HCPs’ perceptions about the barriers and facilitators to the achievement of PA promotional goals?

## 2. Methods

The systematic review was conducted and reported in accordance with the PRISMA (Preferred Reporting Items for Systematic Reviews and Meta-Analyses) Statement [55].

### 2.1. Inclusion and Exclusion Criteria

The study population consisted of all HCPs (e.g., General Practitioners (GPs), Physicians, Nurses, Physiotherapists, Exercise Physiologists (EPs), Dietitians, Diabetes Health Educators, Pharmacists, Surgeons, Podiatrists, Oncologists, Occupational and Physical Therapists and Healthcare Assistants). There was no restriction on study design however, interventional studies (randomized control design and quasi-experimental designs) were excluded given that the aim of this review was to examine the perceptions of HCPs independent of any intervention. Other requirements for inclusion were that studies must have:

1. Included adult participants aged 18 years and above.
2. Considered HCPs’ attitudes or perceptions to PA promotion

Articles were excluded if they did not meet the inclusion criteria and/or they:

1. Considered opinions other than those of HCPs (e.g., patients)
2. Were review papers and
3. Perceptions of HCPs about PA promotion was not specifically discussed.

### 2.2. Search Strategy

Seven electronic databases comprising, Cinahl, Informit, Medline Ovid, Medline (Pubmed), Scopus, SportDiscus, and The Cochrane Library were searched. Peer reviewed primary articles, written in English and published between February 2010 and February 2020 (a decade of literature) were included in this review. The search was limited to a decade in order to facilitate the evaluation of HCPs’ PA promotional practices after the publication of WHO’s 2010 global recommendations on PA for health [3]. Text words and indexed terms like “healthcare practitioner, healthcare professionals, healthcare personnel, primary healthcare personnel, physical activity promotion, health promotion, perceptions, views, perspectives, knowledge, beliefs, attitude, inactivity, physical inactivity and chronic diseases” were included in the search terms. The comprehensive search strategy used for this review is presented in Appendix A. Reference lists from previous reviews and included studies were also screened for additional inclusions.

### 2.3. Study Selection

All the identified articles were imported into Endnote X9 software, then titles and abstracts were screened. Two authors (FAA and BSMA) independently screened the titles and abstracts of the retrieved articles and excluded those articles which did not meet the inclusion criteria. Subsequently, full-text articles categorized as potentially eligible for inclusion were jointly screened by the two authors in a consensus meeting and disagreements were resolved in real time until consensus was reached.

### 2.4. Data Synthesis and Analysis

Meta-analysis was not possible, due to the heterogeneous nature of the included articles [55]. A data extraction form was developed and used to collect relevant information from all the included studies. Descriptive data including author, study year, country of study, study design, type of healthcare professional and participant population, gender and mean age were extracted from each of the selected studies. To explore participants’ perceptions regarding PA promotion, the following key determinant factors to PA promotion were extracted: HCPs’ knowledge of PA, confidence in promoting PA, importance of PA promotion, role in PA promotion, PA assessment, how HCPs currently promote PA, perceived effectiveness of PA promotion, and perceived barriers and facilitators to PA promotion. These factors were adapted from the classifications reported in Fleuren et al. [56] and Chaudoir et al.’s [57] studies on the factors influencing innovations in healthcare.

To categorize the extracted facilitators and barriers from this review, the refined Theoretical Domain Framework (TDF) was adopted [58]. This framework contains 14 domains which are used for coding in behavioral change and implementation research. The TDF domains included: knowledge, skill, social/professional role and identity, beliefs about capabilities, optimism, beliefs about consequences, reinforcement, intentions, goals, memory, attention, decision process, environmental context and resources, social influences, emotion, and behavioral regulation [59]. Two authors (FAA and BSMA) independently extracted and categorized facilitators and barriers from each of the studies. After extracting and categorizing each of these determinants, the two authors met to harmonize the extracted factors as determined by the TDF domain classification. All discrepancies were resolved through discussion and re-examining referenced materials. Identical TDF factors were categorized into sub-themes and domains with multiple themes were deemed crucial TDF domains [60].

For qualitative studies, inductive content analysis was employed [61]. The analysis included three stages of coding, creating categories and abstraction. One author (F.A.A.) extracted data, defined, and developed coding frames for all the key determinant variables in the first stage. Two authors (F.A.A. and B.S.M.A.) designed preliminary categories in the second stage. In the third stage, final categories were developed and labelled by both authors while, all differences were resolved in a consensus meeting. A replication test was used to validate and determine possible extensions to coding frames.

### 2.5. Risk of Bias Assessment

Quality Assessment Tool for Studies with Diverse Designs (QATSDD) was used to assess the methodological consistency of the included studies [62]. This tool contains 16 items and is used for examining studies with different research designs. Each of the included studies was graded on a scale of 0 to 3 for each criterion, with 0 = not at all, 1 = very slightly, 2 = moderately, and 3 = complete. To assess the methodological quality of the each of the included studies, the criteria scores were summed and expressed as a percentage of the maximum possible score. The percentage scores were classified into low (<50%), medium (50–80%) or high (>80%) quality evidence for easy identification. The QATSDD criteria included: (1) theoretical framework; (2) aims/objectives; (3) description of research setting; (4) sample size; (5) representative sample of target group; (6) procedure for data collection; (7) rationale for choice of data collection tool(s); (8) detailed recruitment data; (9) assessment of reliability and validity of measurement tool(s) (Quantitative only); (10) fit between research question and method of data collection (Quantitative only); (11) fit between research question and data collection method (Qualitative only); (12) fit between research question and method of analysis; (13) good justification for analytical method selected; (14) reliability of analytical process (Qualitative only); (15) evidence of user involvement in design; (16) strengths and limitations.

## 3. Results

### 3.1. Included Studies

**Figure 1:**
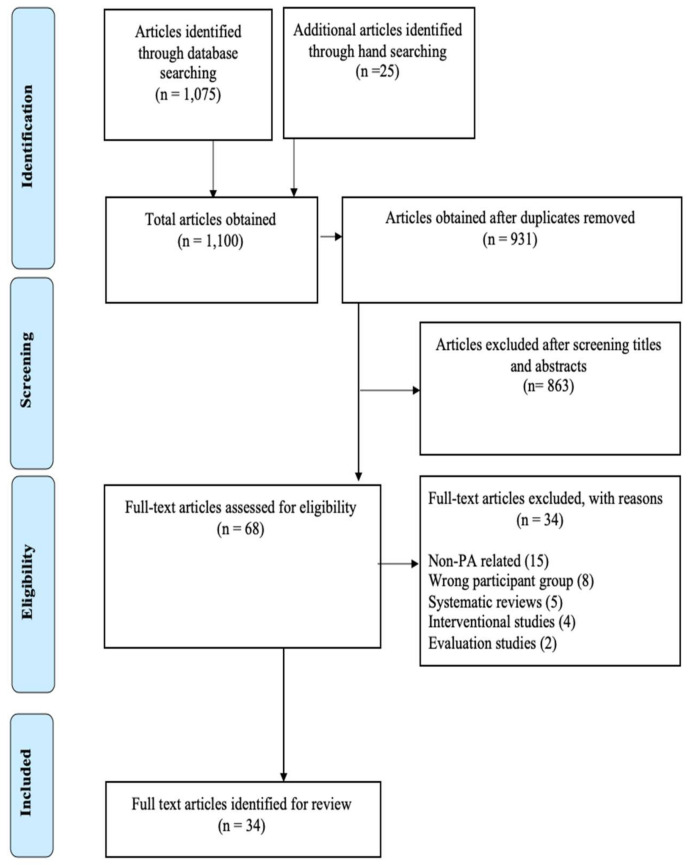
Flow chart of the study selection protocol.

One thousand one hundred (1100) articles were identified from all searched databases and imported into Endnote. After screening the titles and abstracts of the articles identified and reviewing 68 full texts, 34 studies met the inclusion criteria for this review (Figure 1).

### 3.2. Study Characteristics

A summary of the characteristics of the included studies is presented in Table 1. Appendix B provides legends for all the tables in the paper. Twelve (12) of the 34 studies in this review, explored the perceptions of HCPs practising in Europe [53,63,64,65,66,67,68,69,70,71,72,73], 10 were conducted in Australia and New Zealand [31,51,69,74,75,76,77,78,79,80], five each from the UK [52,81,82,83,84] and USA [85,86,87,88,89], two from Africa [75,90], and one from India [91]. The study designs were varied with 20 of the 34 included studies employing a cross sectional design [31,65,66,67,68,70,71,72,73,74,76,79,85,86,87,88,89,90,91,92], the majority of which sampled the opinions of HCPs about PA promotion using questionnaires, 11 studies were qualitative (using semi-structured interviews and focus groups) [52,53,64,69,77,78,80,81,82,83,84] while longitudinal [51], mixed [75], and multi method [63] designs were employed in one study each. Across the 34 included studies, 20 explored the perceptions of a homogenous group of HCPs [51,64,67,68,69,70,72,74,75,76,77,78,79,81,83,84,87,88,89,90] including seven physiotherapists studies [64,69,75,76,83,84,90], five GPs studies [51,67,68,77,78], three physician studies [69,72,89] and one each for psychologists [74], nurses [87], sport medicine physicians [88], physical therapists [79], and healthcare assistants [81]. The other 14 studies explored the perceptions of heterogenous groups of HCPs [30,51,52,63,65,66,71,73,80,82,85,86,91,92]. The HCPs included in these heterogeneous groups included nurses [31,52,53,63,65,66,71,80,82,85,86,92], GPs [52,53,63,65,73,85,91], physicians [53,65,82,86,91], physiotherapists [63,71,73,80,92], dietitians [80,82,92], surgeons [63,80,86], oncologist [31,63], radiation therapists [31,63], occupational therapists [81,92], and only one study explored the perceptions of exercise physiologists [92], pharmacists [92], internists [85], rheumatologists [66], and physical therapists [66].

The total number of participants included in the studies in this review was 11, 862. More males (7,033; 59.3%) compared to females (4, 829; 40.7%) were included in the studies. Participants’ mean age ranged from 30.2 ±9.8 to 59.9 ±5.7 years while the number of participants per study ranged from six (6) to 2,846. HCPs’ perceptions about key determinants of effective PA promotion including: PA knowledge, confidence in PA promotion, PA importance, role in promoting PA, PA assessment, how they promoted PA and the barriers and facilitators to effective PA promotion were extracted from all the 34 studies. The majority of studies (n = 27), recorded the perceptions of HCPs on how PA was promoted [31,51,52,63,64,65,66,67,68,69,71,72,73,74,75,76,79,82,83,84,85,86,88,89,90,91,92]; 26 on barriers to PA promotion [31,52,53,63,64,65,67,68,69,71,73,75,76,77,78,80,81,82,83,84,85,86,88,89,90,92]; 20 on HCPs’ role in PA promotion [31,52,63,64,67,68,69,73,76,79,80,81,83,84,85,87,89,90,91,92]; 15 on HCPs’ knowledge of PA ) [53,65,66,69,70,73,74,76,79,84,85,87,88,91,92]; 12 on the importance of promoting PA [31,52,63,66,74,78,81,84,87,89,90,91]; 11 on HCPs’ confidence in promoting PA [31,69,73,74,76,79,80,82,89,90,92]; nine on facilitators to promoting PA [52,63,64,68,71,78,81,83,85,89]; eight on their assessment of PA [53,73,74,83,86,88,90,91] and three on the effectiveness of promoting PA [74,89,90]. Each of these key determining factors to PA promotion are described in more detail below.

### 3.3. Healthcare Professionals (HCPs)’ Perceived Knowledge of Physical Activity (PA)

HCPs’ perceptions of their knowledge of PA was explored in 44.1% (n = 15) of the included studies (Table 2) [53,65,66,69,70,73,74,76,79,84,85,87,88,91,92]. In 85.7% (n = 12) of these 15 studies, varying percentages of HCPs indicated that they had some form of PA knowledge (12–64.1%). However, GPs, physicians, nurses and rheumatologists from three group studies indicated the need for more training on PA [53,65,66]. Two studies indicated that participants had some university education on PA [74,91].

### 3.4. HCPs’ Perceived Confidence in Promoting PA

HCPs expressed their confidence in promoting PA in 32.3% (n = 11) of the 34 included studies (Table 2) [31,69,74,76,79,80,82,89,90,92]. In over half of the studies (n = 6), 68 to 95.3% of participants indicated that they were confident in promoting PA [31,73,74,76,89,90]. In one of the remaining five studies, EPs and physiotherapists were judged to be more confident than other HCPs in providing general and specific PA advice to patients [92]. Another study indicated that confidence was key to PA promotion and equally, found significant associations between confidence and HCPs’ PA enquiry and advise habits [70]. Lastly, dietitians indicated that their own personal interest in a particular sport and PA habits enhanced their confidence in promoting PA [82].

### 3.5. HCPs’ Perceived Importance of PA and Its Promotion

HCPs’ perceptions about the importance of promoting PA was evident in 35.2% (n = 12) of the studies (Table 2) [31,52,63,66,74,78,81,84,87,89,90,91]. In these studies, 86 to 100% of participants indicated that PA was important for their patients. They agreed that PA could be essential in the management of a myriad of diseases (e.g., rheumatoid arthritis, mental health, respiratory diseases, cancers, and spinal cord injuries), rehabilitation, and the improvement of quality of life. In one of these 12 studies however, only 12% of psychologists noted that PA was important for their patients [74]. In another study, physicians signaled the importance of promoting PA to patients by expressing that patients will value their PA advice [89]. Similarly, GPs affirmed that prescribing PA could be a viable non-pharmacological intervention [78].

### 3.6. HCPs’ Perceived Role in PA Promotion

HCPs’ perceptions about their role in PA promotion was examined in 58.8% (n = 20) of the included studies (Table 2) [31,52,63,64,67,68,69,73,76,79,80,81,83,84,85,87,89,90,91,92]. Between 78% and 97% of HCPs indicated that PA promotion was part of their role in 13 of the 20 included studies [63,64,67,69,73,76,79,83,85,87,89,90,91]. However, HCPs’ views on their part in PA promotion was divergent in seven studies [31,52,68,80,81,84,92]. Four of these seven studies examined the views of heterogenous groups of HCPs while the remaining three examined the views of a homogenous group of HCPs (GPs, healthcare assistants and physiotherapists respectively) [68,81,84]. Across the heterogenous group, some HCPs thought their role in PA promotion was limited and that they should only advise patients on PA when requested [52,68]. Others thought PA should only be promoted when linked to a condition [80], however, nurses (16% of the total participants) were less likely to acknowledge a role in PA promotion [92]. Over half (51.6%) of the HCPs in a cross sectional study nominated physiotherapists as the specialists suited for PA promotion while unspecified exercise specialists was nominated by 46.7% [31]. Healthcare assistants viewed PA promotion as part of their role but were uncertain to what extent [81]. Interestingly, one of the qualitative studies indicated that physiotherapists did not promote PA because they considered it outside their role [84].

### 3.7. HCPs’ Perceptions on PA Assessment

Only eight (23.5%) of the included studies recorded HCPs’ perceptions about their assessment of patients’ PA (Table 2) [53,73,74,83,86,88,90,91]. Across these studies, between 42.5% and 61% of HCPs reportedly assessed patients for PA. However, HCPs in one of the studies indicated that a patient’s physical condition (ability to participate in PA), interest in PA, and former PA lifestyle were three factors informing their decision for PA assessment [53].

### 3.8. HCPs’ Perceptions on how PA Was Promoted

The majority of the included studies 79.4% (n = 27) recorded the perceptions of HCPs on how they promoted PA (Table 2) [31,51,52,63,64,65,66,67,68,69,71,72,73,74,75,76,79,82,83,84,85,86,88,89,90,91,92]. Four categories emerged from HCPs’ views regarding how PA was promoted. They included: those who indicated that they provided verbal advice, written materials (e.g., PA pamphlets or brochures), referral to an exercise specialist (e.g., EPs, physiotherapists or sports medicine specialists), and those who recommended PA interventions to manage the risk factor(s). In 85% (n = 23) of these studies, the majority of HCPs indicated that they promoted PA by providing some form of PA advice and/or counselling [31,51,52,65,66,67,68,70,71,72,73,74,75,76,79,82,83,85,86,88,90,91,92]. Across the homogenous participant group studies, 78–93.8% of physiotherapists, 57–100% of GPs, 88% of physicians, 74% of sport medicine physicians, 54% of physical therapists, and 33% of psychologists indicated that they regularly advised their patients to participate in PA. Across the heterogenous participant group studies, 50.6 to 98% of HCPs noted that they promoted PA to their patients by providing advice or counselling.

Some HCPs in a qualitative study, however, indicated that the advice was targeted at the individuals they perceived would be motivated to change PA habits [52], others claimed they promoted PA based on their experience of a particular sport [82] while three HCPs in a cross-sectional study indicated that they advised their clients not to do PA for unstated reasons [65]. In another study, sport medicine physicians revealed that those who promoted PA by giving written PA prescriptions recorded better improvements in their patients PA levels [88]. In 14.8% (n = 4) of these studies, 73–93.4% of physiotherapists noted that they promoted PA by providing some form of written material [64,73,75,76]. In three different cross-sectional studies, respectively, 24% of physiotherapists [90] and 40% of GPs [67] indicated that they provided written materials to patients while some HCPs stated that giving out written materials was most feasible for them in the third study [92]. HCPs (the majority of which were GPs) across five studies (18.5%) referred patients to exercise specialists for PA intervention [51,63,68,73,86].

Across another five studies, HCPs managed the risk factors (promoting PA via other means outside advice/counselling, providing writing materials and referral to exercise specialists) [69,73,84,86,88]. This group consisted of mainly physiotherapists [69,73,84] and physicians [86,88]. Among the studies that patients were referred to exercise specialists, only in one study did 40% of HCPs indicate that they referred clients to physiotherapists [63], others did not specify the specialists they referred their clients to. Across the five studies reporting the views of HCPs who managed their client’s risks factors, two stated that, about 26% of the HCPs provided written PA prescription [86,88], interventions to change behavior in one study [73], PA seminars in another study [69] while structured gym sessions, group exercises, and recreational sports activities were conducted by physiotherapists in the last study [84].

### 3.9. HCPs’ Perceived Effectiveness in Promoting PA

The perceived effectiveness of PA promotion was recorded in only three studies (8.8%) (Table 2) [74,89,90]. Sixty-six (66) to 93% of the HCPs signaled that they were effective in promoting PA. Each of the three studies explored the perceptions of a single homogenous group of HCPs including: 88% of physicians who indicated that they were effective in promoting PA to their patients [89], 93% of psychologists, who suggested that PA advice and counselling could be vital in psychological treatment [74] and 66% of physiotherapists who reported that PA counselling was effective in promoting PA among their clients [90].

### 3.10. HCPs’ Perceived Barriers and Facilitators to PA Promotion

Table 3 contains a summary of HCPs’ perceptions about the barriers and facilitators to PA promotion respectively. Over seventy six percent (76.5%; n = 26) of the studies included in this review examined participants’ views about barriers to PA promotion [31,52,53,63,64,65,67,68,69,71,73,75,76,77,78,80,81,82,83,84,85,86,88,89,90,92] while only 26.5% (n = 9) of the studies examined participants’ views about facilitators to PA promotion [52,63,64,68,71,78,81,83,89]. Based on the TDF domain classifications, the extracted barriers and facilitators were coded and categorized into themes. Across these studies, 131 data points for barriers and 29 for facilitators to PA promotion were identified. Among all the factors grouped into the TDF domains, environmental context and resource domains ranked highest among HCPs’ perceived barriers with 39 data points [31,52,53,63,64,65,67,69,70,71,73,75,76,77,78,80,81,82,84,85,86,88,89,90,92] whereas reinforcements ranked as the most perceived facilitator to PA promotion with 8 data points [52,63,64,68,83].

Other barrier domains in descending order included: 18 data points for knowledge [63,64,67,70,71,73,76,80,81,82,84,85,88,90,92], 14 each for beliefs about consequences [52,53,71,76,77,81,84,86,92] and social influences [31,64,67,68,69,70,73,80,83,85,89], 10 for skill [64,70,76,81,82,85,86,90,92], eight for optimism [76,81,84,86,92], seven for intentions [64,68,76,83,85,86,92], six for reinforcement [65,76,84,85,88,92], four for emotion [52,71,85,86], three each for beliefs about capabilities [64,77], and social/professional role and identity [69,83,84], two for goals [83,85], and one each for memory, attention, decision process [83], and behavioral regulation [75]. For facilitators, other domains in descending order included: Six data points for environmental context and resources [63,64,68,71,83,89], five for knowledge [63,68,83,89], three for social/professional role and identity [52,68,83], two for social influences [81,83], and one each for skill [52], intentions [64], goals [78], memory, attention, decision process [64], emotions [83], and behavioural regulation [81]. No facilitating determinants were recorded for TDF domains of optimism or beliefs about capabilities and consequences.

### 3.11. Assessment of Methodological Quality

QATSDD assessment indicated that 67.6% (n = 23) of the included studies were medium quality studies [51,52,53,63,65,66,67,69,71,73,74,75,76,78,79,81,82,85,86,87,88,90,92], 17.6% (n = 6) were high quality studies [64,70,77,80,83,84], and 14.7% (n = 5) were low quality studies [31,68,72,89,91] (Table 4). Individual scores ranged from 35.7 to 83.3%. All except one [70] of the top-quality studies were qualitative and were judged to be explicit in their methodology while all the low-quality studies were quantitative and some of the weaknesses identified from these studies included: lack of theoretical framework, inadequate sample sizes and poor reliability. Five out of the six high quality studies recorded the opinions of homogenous groups of HCPs including three physiotherapists studies [64,83,84] and one each of physician [71] and GP studies [78]. The findings across the high quality studies indicated that though HCPs (mostly physiotherapists, physicians, and GPs) currently promote PA, additional training and support are required to effectively promote PA and including patients in the structuring and implementation of PA interventions could enhance the achievement of PA promotional goals. The five low quality studies included two physician studies [72,89], two heterogenous HCP studies [31,91], and one GP study [68]. There was no notable difference between the findings from the high and low quality studies.

## 4. Discussion

This systematic review explored and synthesized the perceptions of HCPs about key factors influencing effective promotion of PA. This review has highlighted pertinent issues including increased workload and time pressure on frontline HCPs such as GPs in the promotion of PA. The underutilization of the services of PA specialists such as EPs is also highlighted though, these specialists are more suitable for specific and top-level PA support services which majority of the population perhaps seek when specialist PA requirements are indicated. This is evident from the insights provided by the HCPs in relation to how they promote PA with most HCPs viewing inadequate counselling time as a major barrier to PA promotion. These findings corroborate the work of other studies [52,93]. For example, Hebert et al. [93] indicated that HCPs are open to the view of PA promotion, however, personal, and organizational obstacles might prevent effective integration of PA promotion into primary care. This was further reiterated by Din et al. [52], who concluded that barriers to PA promotion including expertise and time limitations should be resolved in order to facilitate HCPs’ ability to promote PA. Given that patients value the advice of HCPs, a concise and strategic behavioral change intervention delivered by HCPs might be useful in enhancing PA and reducing inactivity [94]. Consequently, continuous training for HCPs, the adoption of PA prescription and referral programmes as universal standard treatments and the integration of PA and healthcare services might enhance individual levels of PA and help meet the WHO goals for the reduction of inactivity, morbidity, and mortality [95].

HCPs’ reported knowledge of PA and its promotion pathways were quite varied, and this could be an indication that more awareness and training may be required. This finding was evident in the study by Cantwell et al. [63] who indicated that HCPs can provide crucial PA prompts to patients but may lack the requisite knowledge to give explicit PA advice. Given that the findings from this review include an assortment of HCPs’ opinions, it therefore provides an extension to the work of Cantwell and colleagues who explored the perceptions of mainly oncology specialists. Factors making up three domains of TDF (knowledge, skills, and reinforcement) signaled the major impact knowledge has on HCP’s propensity to promote PA. Jones et al. [96] suggested that ongoing training and the employment of evidenced-based practice to promote PA or refer cases to PA specialists could be helpful.

Despite the divergence in knowledge, there were optimistic views from HCPs about the importance of promoting PA, their effectiveness and confidence in promoting it. For example, the majority of physicians, psychologists and physiotherapists from a cross-sectional study, indicated that they were confident in promoting PA and perceived PA to be effective. Additionally, all except psychologists considered PA to be important for their patients. The low perception of the importance of PA among psychologists could be due to paucity of knowledge about the benefits of PA among this group of HCPs required to inform positive behavioral change towards PA promotion [74,97]. HCPs’ views regarding their assessment for PA were inconclusive. Their views suggested that several factors impacted on their decision for PA assessment, and as a result there might be a need for consensus on standard PA assessment procedures [98]. This could enhance evidence-based practice, inform the need for timely PA intervention for chronic diseases, and improve the quality of care and outcome for patients’ conditions.

The majority of HCPs viewed PA promotion to be part of their role. Despite this overwhelming agreement, some HCPs in a qualitative study thought their role was limited and the number of nurses viewing PA promotion as part of their role were less compared to other HCPs across the included studies. When asked about whose role is best suited for promoting PA, HCPs in a cross-sectional study ranked physiotherapists highest and the least were other unspecified PA specialists. However, Williams et al. [84] argued that physiotherapists failed to promote PA because they viewed it outside their role. Possible reasons for this could be because PA promotion is not an integral component of most physiotherapists practice and these specialists now practice with a wider scope which perhaps leads to more divergence in their PA promotional roles [76,99]. Another group of eligible PA specialist who could be best suited for this role are EPs, although their valuable skills in PA promotion are probably underutilized [100,101]. EPs emerge as the best option by virtue of their training in the delivery of clinical PA interventions for the prevention and management of chronic and complex disease conditions [102]. Contrary to expectation, only one of the included studies explored the perceptions of EPs. This could be because exercise physiology is still an evolving profession and EPs are not within the context of the healthcare systems in most countries. Hence, some HCPs like GPs might have limited understanding of how to refer to EPs [103]. Consolidating the valuable access of frontline HCPs like GPs with the PA expertise and extended consultation time of PA specialists like physiotherapists and EPs, could perhaps be the catalyst for the achievement of PA goals [102]. For the wider public without chronic or complex diseases or with limited accessibility to PA specialists, ongoing community-based PA support programmes could be helpful. Hence, further studies into the effectiveness of PA promotional interventions provided by EPs, the functionality of community-based PA support programmes and reasons for weak referral pathways between key healthcare gatekeepers such as GPs and PA specialists such as EPs will be highly valuable.

HCPs’ perceptions about the factors within the TDF domains for barriers and facilitators to PA promotion revealed that achievement of PA promotion goals could be improved by minimizing identified obstacles and boosting the enabling factors. The obstacles included inadequate consultation time and paucity of knowledge about the importance of PA and its promotional pathways, while the facilitators were incentives for key frontline HCPs, providing further training on PA and access to PA educational materials. Addressing these factors, therefore, could enhance HCPs’ knowledge, effectiveness, readiness, and confidence in promoting PA [96]. HCPs viewed “limited consultation time” as the greatest barrier within the environmental context and resources domain of TDF. Based on this result, referral of identified clients to PA specialists for prolonged and effective PA consultations could be a remedy [100,104]. For example, one of the significant findings from the study by Freene et al. [92] and echoed by O’Brien et al. [105], indicated that physiotherapists and EPs were more confident in providing PA advice to patients. Hence, indicating that these PA specialists can be key players in interventions designed to combat complex and chronic diseases. In view of the perceived facilitating TDF factors, the gains of PA promotion could be enhanced if all the potential pathways for PA promotion are utilized to their full capacity.

In summary, the general perceptions of HCPs about key determinants of PA promotion are encouraging. Current PA promotion practices could be made more efficient if fundamental obstacles such as limited consultation time, underutilization of PA referral services and the lack of PA knowledge and resources are addressed.

### 4.1. Implications for Practice and Research

The evidence from this review could inform future research on improving the integrative health promotional practice in healthcare settings. It could also be translated into evidence-based practice for PA promotion in healthcare settings. To facilitate the translation of research into practice, stakeholder networks could be established to train, encourage and enforce PA promotional goals for sustainable and enhanced patient health outcomes. The key PA determinants identified in this review can be used to educate and enhance the PA knowledge of frontline HCPs like GPs. Particularly, the unique expertise of different PA specialists and the varied roles they can play in the effective delivery of optimum healthcare services can be further emphasized. Stakeholders can also utilize the findings in this review to plan, implement and evaluate PA promotional interventions in healthcare settings. Future studies that focus on modifying HCPs’ PA habits and promotion practices as well as strengthening referral pathways between key healthcare gatekeepers such as GPs and PA specialists will be helpful. Given that QATSDD assessment indicated that most of the included studies lacked theoretical framework, future studies could be structured to relevant theoretical framework in order to enhance the quality of the study.

### 4.2. Strength and Limitation

This is the first systematic review on PA promotion that explored the perceptions of varied HCPs regarding key determinants to PA promotion. The results from this review could strengthen the evidence-base for research on ways to enhance sustainable PA promotion among HCPs. Employing the TDF behavioral domain framework and assessing the quality of the included studies, further strengthened the evidence in this systematic review. QATSDD assessment indicated that almost two-thirds of the studies were medium quality studies. The studies were judged to be strong in their aims and objectives and the methods used for recruitment, collection and the analysis of data. The studies, however, lacked or failed to explicitly describe relevant theoretical frameworks, research questions and sample sizes. Other limitations of this review include the heterogeneity of the included studies and the exclusion of some relevant studies due to pre-set inclusion criteria such as the selection of English language studies only. The distinction in the training programmes, role description, and expertise of various HCP professions across the globe might have impacted on study results as well.

## 5. Conclusions

The findings of this review revealed that the optimum utilization of all PA promotion pathways (Advice/counselling, provision of PA resources or prescription, and the onward referral to PA specialists such as EPs) and addressing key TDF domain factors could be the potential turning point in a bid for sustainable solutions to the success of PA promotional goals. There is an accessible PA expertise for the non-pharmacological prevention and treatment of chronic diseases within healthcare systems, though this pathway is currently underutilized. Hence, an effective framework for HCPs’ behavioral modification and the enhancement of collaborative interdisciplinary care for chronic and complex disease management will be invaluable. This was echoed by the WHO, where they indicated that HCPs are crucial to the success of PA promotion. Ultimately, development of functional stakeholder networks for training, promotion, implementation, and the evaluation of PA promotional goals could offer sustainable solutions and improved health outcomes for patients.

## Figures and Tables

**Table 1:** Study characteristics.

Author, Study Year & Reference	Country of Study	Study Design	Type of Healthcare Professional(s) (HCPs)	Participants (No., Gender, Mean Age [yrs.])
Spellman et al. 2014 [31]	Australia	Cross sectional study	HCPs (Clinicians i.e., Urologists*; Medical Oncologists*; Radiation Oncologists* and Nurses*)	N = 31; Females (29%, n = 9); Age: (43.5 ± 16.2)
Barnes et al. 2019 [51]	Australia	Longitudinal study	GPs	N = 2846; Females (20%, n = 570); Age: (49.8 ± 4.08)
Din et al. 2015 [52]	UK	Qualitative study	HCPs (GPs – 67.3%, Nurses – 19.5% & Practice Managers – 13.0%)	N = 46; Females (56.5%, n =26) Age: *
Haussmann et al. 2018 [53]	Germany	Qualitative study	HCPs (GPs – 33.3%; Physicians – 33.3% & Nurses – 33.3%)	N = 30; Females (63%, n = 19); Age: (45.0 ± 11.5)
Cantwell et al. 2018 [63]	Ireland	Multi-methods (Delphi method)	HCPs (Nurses – 48%; Oncologists – 27%; GPs – 8%; Surgeons – 19.5%: Physiotherapists – 2% & Radiation therapists – 0.5%)	N = 91; Females (67%, n = 61); Age: (44.02 ± 15.6)
Eisele et al. 2020 [64]	Germany	Qualitative study	Physiotherapists	N = 9; Females (66.6%, n = 6); Age: (39 ± 12.0)
Haussmann et al. 2018 [65]	Germany	Cross Sectional Study	HCPs (GPs – 17.2%; Physicians – 40.5% & Nurses – 42.3%)	N = 917; Females (61.3%, n = 563); Age: (30.2 ± 9.8)
Hurkmans et al. 2011 [66]	The Netherlands	Cross Sectional Study	HCPs (Rheumatologists – 34.2%; Nurse – 35.6% & Physical Therapist – 30.2%)	N = 370; Females (66.7%, n = 247); Age: (46 ± 6.0)
Jorgensen et al. 2012 [67]	Denmark	Cross Sectional Study	GPs	N = 223; Females (50.7%, n = 113); Age: (53.4 ± 8.0)
Leemrijse et al. 2015 [68]	The Netherlands	Cross Sectional Study	GPs	N = 340; Females (41.1%, n = 140); Age*
Mulligan et al. 2011 [69]	New Zealand & Sweden	Qualitative study	Physiotherapists	N = 9; Females (88.8%, n = 8); Age**
O’Brien et al. 2019 [70]	Ireland	Cross Sectional Study	Physicians	N = 595; Females (56.3%, n = 335); Age: (42.6 ± 12.1)
Sassen et al. 2011 [71]	The Netherlands	Cross sectional study	HCPs (Nurses– 40% & Physiotherapists – 60%)	N = 278; Females (73.7%, n = 205); Age: (36.2 ± 10.1)
Suija et al. 2010 [72]	Estonia	Cross sectional study	Physicians**	N = 198; All Females; Age: (47.1 ± 9.4)
Barrett et al. 2013 [73]	Ireland	Cross-sectional study	GPs and Physiotherapists	N = 431; Females (43.4%, n = 187); Age: *
Burton et al. 2010 [74]	Australia	Cross-sectional study	Psychologists	N = 236; Females (84.7%, n = 200); Age: (42.12 ± 11.03)
Frantz & Ngambare 2013 [75]	Rwanda	Mixed Methods	Physiotherapists	N = 92; Females (30%, n = 28); Age: (32.49 ± 6.56)
Freene et al. 2017 [76]	Australia	Cross Sectional Study	Physiotherapists	N = 257; Females (77.8%, n = 200); Age: (43.2 ± 15.4)
Patel et al. 2012 [77]	New Zealand	Qualitative study	GPs	N = 15; Females (66.6%, n = 10); Age: (50.8 ± 7.1)
Patel et al. 2011 [78]	New Zealand	Qualitative study	GPs	N = 15; Females (66.6%, n = 10); Age: (50.8 ± 7.1)
Shirley et al. 2010 [79]	Australia	Cross sectional study	Physical Therapy practitioners	N = 318; Females (73%, n = 233); Age: (41.8 ± 9.4)
Speake et al. 2019 [80]	Australia	Qualitative study	HCPs (Clinical specialists in pain management*; Occupational therapists*; Nurses* (Continence and diabetes), Consultants in diabetes* and Orthopaedics*; Specialist diabetes dieticians*; Physiotherapists* (continence and MSK), specialist and advanced* Podiatrists)	N = 22; Females (68.1%, n = 15); Age*
Kinnafick et al. 2018 [81]	UK	Qualitative study	Healthcare Assistants	N = 11; Females (54.5%, n = 6); Age: (30.27 ± 7.75)
Litchfield et al. 2019 [82]	UK	Qualitative study	HCPs (Dietitians – 33.3%; Specialist physicians – 16.6%; Nurses – 50%)	N = 6; Females*; Age*
Lowe et al. 2018 [83]	UK	Qualitative study	Physiotherapists	N = 12; Females (58%, n = 7); Age*
Williams et al. 2018 [84]	UK	Qualitative	Physiotherapists	N = 18; Females (72.2%, n = 13); Age**
Courtney-Long et al. 2017 [85]	USA	Cross-sectional study	HCPs (Family/GPs – 44.1%; Internists – 37.1% & Nurse practitioners – 18.9%)	N = 1760; Females (38.5%, n = 678); Age: (59.9 ± 5.7)
Omura et al. 2018 [86]	USA	Cross Sectional Study	HCPs (Physicians – 57.1%; Paediatricians – 14.2%; Obstetrician & Gynaecologists – 14.2% & Nurses – 14.3%)	N = 1751; Females (14.5%, n = 254); Age: (51.5 ± 11.5)
Pearson et al. 2018 [87]	USA	Cross sectional study	Nurses	N = 111; Females (78.3%, n = 87); Age: (36.8 ± 11.9)
Pojednic et al. 2017 [88]	USA	Cross sectional study	Sport Medicine Physicians	N = 412; Females (47%, n = 194); Age: (47.1 ± 2)
Tucker et al. 2017 [89]	USA	Cross sectional study	Physicians**	N = 25; Females (64%, n = 16); Age**
Abaraogu et al. 2016 [90]	Nigeria	Cross-sectional study	Physiotherapists	N = 103; Females (30%, n = 31); Age: (34.5 ± 9.5)
Patra et al. 2015 [91]	India	Cross sectional study	HCPs (GPs– 32.8% & Physicians – 67.2%)	N = 146; Females (41%, n = 60); Age: (43 ± 11.3)
Freene et al. 2019 [92]	Australia	Cross Sectional Study	HCPs (Physiotherapists – 59%; Nurses – 16%; Exercise Physiologists – 13%; Occupational therapists – 6%; Dietitians – 3% & Pharmacists – 2%)	N = 433; Females (79%, n = 340); Age: (41.7 ± 15.3)

*= Item not indicated ** = Values/categories not specified.

**Table 2:** Participants’ perceptions about physical activity (PA) knowledge, confidence, importance, role assessment, recommendation, and effectiveness.

Author, Study year & Reference	Knowledge of PA	Confidence in Promoting PA	Importance of PA and its Promotion	Role in PA Promotion	PA Assessment	How PA was Promoted	Perceived Effectiveness of PA Promotion
Spellman et al. 2014 [31]		83.9% (n=26; M=4.2, SD=0.76) of the HCPs agreed or strongly agreed that they were confident in providing general advice about physical activity to prostate cancer survivors	Almost all the HCPs (n=29, 93.6%; mean (M)=4.52, standard deviation (SD)=0.51) strongly agreed or agreed that regular physical activity can improve cancer patients’ quality of life	On who’s role to recommend PA, over 50% of HCPs (n=16) nominated a Physiotherapist; followed by a Urology Nurse (n=14, 46.7%) and an Exercise Specialist (n=14, 46.7%)	NA	On recommended PA, 3.2% (n=1) of HCPs always gave advice; 36% (n=11) often gave advice, 45.2% (n=14) sometimes gave advice and 16% (n=5) rarely gave advice, 80.8% (n=25) recommended cardiovascular PA followed by weights training. On how PA was recommended; all participants (n=31) gave advice verbally and 9.7% (n=3) provided pamphlets to their patients. No participant reported the referral of patients to an exercise specialist	NA
Barnes et al. 2019 [51]	NA					GPs provided Physical Activity Management (PAM) for 58, (2.0%) prostate cancer survivors. The PAM provided was physical activity counselling on 39 and a physical activity referral on 19 occasions.	NA
Din et al. 2015 [52]	NA		Most HCPs acknowledged the importance of promoting physical activity in order to improve public health	HCPs commonly saw their role as giving advice when asked for, rather than ‘coercing’ patients into changing their behavior		HCP’s selectively provided PA advice to patients. The advice was targeted at individuals they felt would be motivated to change. Such judgements were sometimes based on patients’ physical appearance, conditions, age and/or gender	
Haussmann et al. 2018 [53]	HCPs expressed their wish for more PA related information for themselves and their patients with cancer	NA			The perceived patient characteristics influencing HCPs impression for PA assessment were patients’ physical condition (n = 16), patients’ assumed interest in PA (n = 11) and patients’ former PA lifestyle (n = 10).	NA	
Cantwell et al. 2018 [63]	NA		Over 86% of HCPs agreed or strongly agreed that PA has so many health benefits and can improve quality of life	Almost 88% of HCPs either agreed or strongly agreed that discussing physical activity with cancer patients was part of their role	NA	PA was frequently recommended to myriads of cancer patients depending on the stage of cancer. PA promotion was given verbally or by a referral to a physiotherapist or exercise specialist (40%) or referral to a community-based programme (28%)	NA
Eisele et al. 2020 [64]	NA			Physiotherapists indicated that they felt responsible to instruct PA. Some also regarded it as their role to advice and motivate patients on routine PA implementation		Some physiotherapists design home-based PA for their clients (goal setting behavior). Client’s choice of activities is considered by some physiotherapists while others do not	
Haussmann et al. 2018 [65]	69.5% of all HCPs requested at least one offered PA information resource. 53.5% requested a booklet, scientific paper by 53.0%, and educational course by 27.6%.	NA				PA was recommended often or routinely in 88.5% of physicians working in outpatient care, 78.1% of physicians working in inpatient care, and 73.1% of oncology nurses. Three HCPs (indicated that they advised against doing PA	NA
Hurkmans et al. 2011 [66]	54% (n = 68) of Rheumatologists indicated that they were interested in additional education on the promotion of PA compared to Clinical Nurse specialists (n = 112, 85%) and Physical therapists (n=81, 72%) (both *p* < 0.001)	NA	Majority of Rheumatologists (n=118, 94%), Clinical Nurse specialists (n=132, 100%), and Physical therapists (n = 109, 100%) agreed that obtaining and/or maintaining a sufficient level of PA is an important health goal in the management of patients with rheumatoid arthritis	NA		86% (n = 107) of Rheumatologists gave advice on PA, 95% of Clinical Nurse specialists (122) and 99% of Physical therapists (n = 109). More Clinical Nurse specialists (n = 52, 41%) and Physical therapists (n = 54, 49%), use the public health recommendation on moderate-intensity PA for patients’ advice	
Jorgensen et al. 2012 [67]	NA			80.2% of GPs reported that promoting PA was one of their work tasks while 19.8% (43/217) did not perceive it as their job or were in doubt	NA	57% of GPs promoted PA daily, 38.6% weekly and 4.5% monthly or seldomly. In about 60% of cases GPs gave PA advice with recommended exercise type, duration, frequency, and intensity and in 40% of cases PA promotion included written material	
Leemrijse et al. 2015 [68]	NA			Half of the GPs thought that they had an important role in stimulating physical activity, while the other half considered their role present but ‘limited	NA	All GPs said they recommend PA to their patients. This was given when PA was relevant for the patients’ health problem or health status. About 70% of GPs referred patients for PA interventions	NA
Mulligan et al. 2011 [69]	Physiotherapists have developed knowledge on and were aware of and supported current national health policies toward PA enhancement	NA		Physiotherapists perceived that they had moved away from what they considered traditional physiotherapy practice and now practiced with a wider scope.	NA	Educational seminars provided opportunities for people with neurological conditions to support and learn from one another while building efficacy and acquiring strategies to take responsibility for their own future health and well-being	
O’Brien et al. 2019 [70]	64.1% of physicians indicated the correct weekly levels of PA recommended by the National PA Guidelines for Ireland. 29% of participants accumulated 4 h of PA promotion training.	Confidence was identified by physicians as an important factor in undertaking PA counselling activities, with a significant association between confidence and PA enquiry and counselling activities observed	NA			88.0% of physicians enquired about PA levels and 86.2% provided verbal PA counselling to at least some of their patients. The majority of participants reported that they did not provide either written advice (82.6%) or PA prescriptions (89.3%)	
Sassen et al. 2011 [71]	NA					56.8% of the HCPs encourage PA among cardiovascular patients.	
Suija et al. 2010 [72]	NA					Physicians claimed that they counsel over 94% of their patients about physical activity	
Barrett et al. 2013 [73]	Below 1/3 of GPs and 1/2 of PTs reported the correct PA guidelines. More PTs compared to GPs significantly recalled the PA guidelines (PTs - 50.5%, n = 45; GPs – 28%, n = 97; *p* < 0.005; χ^2^ = 16.56; df = 1) while 58% (n = 197) of GPs and 37% (n = 33) of PTs were unsure about the frequency of PA.	Seventy two percent (72%, n =247) and 92% (n = 82) of PTs noted that they were confident in providing PA advice to their patients	NA	Ninety five percent (95%) of both GPs (325) and PTs (85) perceived PA promotion to be part of their role	About 41%, (n = 139) of GPs reported opportunistic screening of patients, 37% (n =126) screened patients for PA if relevant to patients’ presentations and 8% (n = 28) routinely screened all their patients for PA. For PTs, 34% (n = 30) screened all patients for PA, 28% (n = 25) screened patients if related to presentations and 24% (n = 21) reported opportunistic screening	Education and advice (GPs = 76%, n = 258 and PTs 97%, n = 86); referrals to other services by GPs (practice nurse = 16%, n = 56; exercise specialist = 14%; n = 49; exercise prescription schemes = 11%; n = 37, gyms = 44%; n = 150). PTs utilised written materials (73%; n = 65), exercise diaries (57%; n = 51), follow up appointments (53%; n = 47) and behavioural modification (GP = 50%, n = 172; PT = 53%, n =47)	NA
Burton et al. 2010 [74]	12% (n=29) of psychologists said they had received PA advice or counselling instructions during undergraduate, 31% (n=61) during postgraduate training and 18% (n = 41) during a workshop/seminar	Over 80% of psychologists were confident to provide general activity advice, discuss options, identify and manage barriers to activity	12% of psychologists (n = 29) strongly agreed/agreed that PA was important for preventing chronic health problems	NA	61% (n = 142) of psychologists reported always asking about PA in the past month	59% of psychologists (n = 139) always discussed PA, 53% (n=22) recommended PA, 33% (n = 77) always gave PA advice	93% (n = 220) of psychologists strongly agreed/agreed that PA advice and counselling could be a useful component of psychological treatment
Frantz & Ngambare 2013 [75]	NA					PA counselling was the predominant health promotions strategy used by physiotherapists (98%) while 9% used written materials.	
Freene et al. 2017 [76]	On general knowledge regarding PA, physiotherapists recommended the following: taking the stairs by 54%; 30 min walk on most days by 43%; exercise that makes you puff and pant by 32%; several 10 min shot walks on most days by 78%	95.3% of physiotherapists indicated that they would feel confident in giving general PA advice to patients while 93% of participants indicated that they would feel confident in suggesting specific PA		Over 97% of physiotherapists indicated that some parts of their role to patients are: discussing the benefits of PA, suggesting ways to improve PA and also acting as PA role model		93.8% of physiotherapists practiced brief PA during consultations with patients, separate one-on-one consultations was practiced by 52.5% of participants, group sessions by 67.3% and distribution of resources (such as brochures) by 93.4% of physiotherapists	
Patel et al. 2012 [77]	NA						
Patel et al. 2011 [78]	NA		GPs perceived Green Prescription to be a beneficial PA tool for drug-free therapeutic processes and health gain, endorsed by them and presented in the same format as prescription medication	NA			
Shirley et al. 2010 [79]	Only one third of the respondents could name the national physical activity recommendation for Australian adults	Physical therapists who gave more patients physical activity advice were more likely to feel confident in suggesting specific physical activity programmes	NA	In both groups, almost all physical therapists thought it should be part of their role to give their patients physical activity advice	NA	Above half of the physical therapists (54%) reported that they encouraged 10 or more patients each month to lead a more physically active lifestyle	
Speake et al. 2019 [80]	NA	HCPs found it challenging to give advice that reflected individual differences. In particular they perceived a need for lower entry points to PA interventions that might be more palatable for their most inactive patients		There was a lack of consensus about roles and responsibilities for promoting PA. HCPs indicated that their primary role was to focus on their patient’s physical health and the specialty of their training. Bringing PA into consultation, had to be linked to the health condition	NA		
Kinnafick et al. 2018 [81]	NA		All health care assistants agreed that exercise was beneficial for patients’ physical and mental health	health care assistants agreed that PA promotion was part of their role, but the extent to which they should encourage PA was unclear to them	NA		
Litchfield et al. 2019 [82]	NA	Dietitians were comfortable presenting advice to individuals who consistently indulge in PA like running or cycling. The standard at which Dietitians exercised or played sport informs the confidence in promoting PA	NA			HCPs promoted PA based on their personal experiences of a particular sport or activity	
Lowe et al. 2018 [83]	NA			Physiotherapists role in educating patients on PA came through strongly as a means of supporting self-management	Physiotherapists integrated PA questions into the subjective assessment and specifically into the social history of their patient	Physiotherapists promoted PA by providing brief advice, brief intervention, cognitive behavioral therapy, and motivational interviewing to their clients	
Williams et al. 2018 [84]	PA knowledge was gained through the practical experience of caring for people with Spinal Cord Injuries (SCI) over time. Participants revealed that: they were not educated on PA during their degree course and had a limited range of other sources of PA knowledge. The value of PA was learnt through seeing the perceived detrimental effects of physical inactivity.	NA	Physiotherapists recognized the value that PA played both in Spinal Cord Injury (SCI) rehabilitation and upon discharge to the community. They drew upon PA in rehabilitation to improve balance, flexibility, strength, and cardiovascular fitness with the aim to improve function and independence	One reason why most of the Physiotherapists failed to promote PA was because it was deemed not to be part of their role	NA	Physiotherapists promoted PA for SCI rehabilitation by providing structured gym sessions and group exercises and recreational sport activities organized by other health professionals	NA
Courtney-Long et al. 2017 [85]	53.7% of HCPs knew the guideline on aerobic activity applied to adults with disabilities with the percentage been highest among those who strongly agreed they felt prepared (62.0%)	NA		About 79% of HCPs or somewhat agreed they felt prepared to recommend physical activity to their patients with disabilities		About 50.6% of HCPs reported recommending PA to patients with disabilities at most clinic visits	
Omura et al. 2018 [86]	NA				Discussing PA with at-risk patients was higher among non-Hispanic HCPs compared to others.	92% of HCPs who encouraged their clients to increased PA used counselling, 78.7% by assessing PA levels, 41.5% provided educational materials, 25.6% by written exercise prescription and 15.1% referred patients for PA interventions.	
Pearson et al. 2018 [87]	In the perceived behavioral control category, high mean scores were noted regarding knowledge, skills and intention to promote PA		The item with the highest mean score (i.e., most agreement with the statement) was PA increases activity tolerance. Mean attitude scores were lowest related to PA causes harm	Mean scores were noted to be highest in the subjective norm category in regard to promotion of PA being a priority of nursing and that engaging patients in PA is the responsibility of RNs.	NA		
Pojednic et al. 2017 [88]	Physicians were most familiar with four activities: walking, aerobic activity, strength training and cycling. 37% of physicians used Exercise is Medicine resources, 19% used tools created individually by clinicians drawn from the internet, books, or elsewhere, and 12% used American Heart Association resources	NA			49% of physicians included PA assessment as a vital sign.	About 74% of physicians recommended and talked about PA 26% provided a written PA prescription. Physicians who provided a written PA prescription reported seeing more improvement in their patients’ physical activity levels	NA
Tucker et al. 2017 [89]	NA	Physicians expressed confidence in their ability to counsel their patients to engage in adequate amounts of PA (68% strongly agreed, 24% somewhat agreed)	Approximately 88% of the Physicians agreed that patients were more likely to adopt healthier lifestyles if their healthcare providers counselled them to do so (44% strongly agreed, 44% agreed somewhat)	88% of Physicians strongly agreed that it was their responsibility to promote a PA, weight loss and healthy weight maintenance among their patients	NA		Most Physicians at least somewhat agreed that they were effective in encouraging patients to engage in health-promoting PA (44% strongly agreed, and 44% somewhat agreed)
Abaraogu et al. 2016 [90]	NA	Over 90% (n = 93) of physiotherapists rated themselves confident in assessing physical inactivity	Counselling patients on PA was considered very important by 87%; (n = 90) of physiotherapists	Addressing physical inactivity was considered high priority and a normal clinical role by 82% (n = 84) of physiotherapists	56%; (n = 58) of physiotherapists ‘‘always’’ or ‘‘usually’’ assess their client’s PA profile	PA was promoted by providing advice; written materials; referral; and managing risk factors. Over 78% regularly advised clients on PA while about 24% gave written PA advice	66% (n = 68) of physiotherapists believed that their counselling is effective
Patra et al. 2015 [91]	24.7% of HCPs reported that they had attended classes on PA in medical college and 26% received formal training for PA counselling	NA	81% of HCPs perceived PA to be important. 52% perceived PA to be beneficial in depression, 22.6% in chronic respiratory disease and 19.8% in cancers	78% of HCPs agreed that PA has a role in primary, 91% in secondary prevention of chronic diseases and a beneficial role in the prevention of heart diseases, obesity and diabetes	42.5% HCPs reported that they always ‘asked’ their patients about their current PA levels	46.6% HCPs always gave ‘verbal advice’. 25.3% always asked and advised’ their patients regarding PA.	NA
Freene et al. 2019 [92]	All HCPs felt they had the skills to promote PA	PTs and EPs were more confident giving general (*p* < 0.001) and specific (*p* < 0.001) PA advice to patients	NA	All HCPs agreed that providing PA advice was part of their role, although nurses were less likely to agree	NA	Brief counselling (n = 392, 91%) and giving out brochures (n = 404, 93%) were reported to be the most feasible methods for PA promotion by HCPs	

NA—Not Available; HCPs—Healthcare Professionals; PA—Physical Activity; SCI—Spinal Cord Injuries; PCPs—Primary Care Providers. * Patel et al. 2012 was not included in the table, because participants did not record their perceptions on any of the factors contained in Table 2.

**Table 3:** Participants’ perceived barriers and facilitators to PA promotion.

TDF Domains	Rank (B - Barrier and F-Facilitator)	Barrier Constructs & Reference Numbers	Facilitator Constructs & Reference Numbers
TDF domain 1: knowledge (This is the recognition that something exists)	B2; F3	Lack of knowledge or training on PA: [64,70,76,81,82,85,86,90,92]	Providing education to HCP’s and patients about the benefits of PA and available promotional programmes: [63]
		Lack of knowledge of PA & promotional pathways: [63,67,70,71,73,80,84,86,88]	Assessable resources on PA promotional programmes (e.g., smart phone apps, assessment tools etc): [63,68,83,89]
TDF domain 2: skill (This is talent acquired by repeated practice)	B5; F5	Lack of knowledge or training on PA: [64,70,76,81,82,85,86,90,92]	Providing supportive and individualized PA programmes: [52]
		Lack of motivational skills to encourage participants: [64]	
TDF domain 3: social/professional role and identify (This is the logical sequence of character exhibited by a person)	B10; F3	Perception of limited role in PA promotion: [84]	Professional collaboration among HCPs: [68,83]
		Lack of cooperation among HCP’s: [69,83]	HCPs physical active lifestyles: [52]
TDF domain 4: beliefs about capabilities (This is the honest and rational acceptance of a particular talent or expertise that can be useful to an individual)	B10; F(Non)	Language barrier: [64]	Not indicated
		Lack of motivational skills to encourage participants: [64]	
		Lack of confidence in promoting PA: [77]	
TDF domain 5: optimism (This is the conviction that an event will occur, or an expected aim will be achieved)	B6; F(Non)	Perceived feeling that PA advice will not convince patient to change behavior: [76,86,92]	Not indicated
		Perceived feeling that PA will not be beneficial for patient: [76,81,84,86,92]	
TDF domain 6: beliefs about consequences (This is the act of embracing the honest and rational result of a particular conduct in a certain circumstance)	B3; F(Non)	Perceived fear of liability and litigation: [52,71,86]	Not indicated
		Perceiving investments in PA promotion to be a misuse of government funds: [52]	
		Perceived feeling that PA advice will not convince patient to change behavior: [76,86,92]	
		Perceived feeling that PA will not be beneficial for patient: [76,81,84,86,92]	
		Perception that PA could be counter-productive: [53,77]	
TDF domain 7: Reinforcements (This is an enhancement of the likelihood of reaction by organizing a conditioner connection between the reaction and the stimulus)	B8; F1	Lack of remuneration or incentives: [65,76,84,85,88,92]	Reported beneficial outcomes of PA: [63,68]
			Repeat appointments with patients: [83]
			Feedback to HCPs on patient’s progress in a programme: [63]
			Affordability of PA and referral pathways services: [68]
			Positive feedback from other patients on PA referral: [52,64]
			Financial incentives to patients: [52]
TDF domain 8: Intentions (This is the deliberate resolve to perform an act in a particular manner)	B7; F5	Patient’s comorbidities or other immediate health issues: [83,85]	Self-motivation and interest by patient to participate in PA: [64]
		Patient’s reduced health status: [68]	
		Prioritizing other interventions: [83]	
		Feeling uncomfortable/inappropriate to speak to patient about PA: [85]	
		Not interested in promoting PA: [76,92]	
TDF domain 9: Goals (This is the intellectual depiction of results that one desires to attain)	B12; F5	Patient’s comorbidities or other immediate health issues: [83,85]	Pre-existing indication for PA intervention: [78]
TDF domain 10: Memory, Attention, Decision process (This is the capacity to keep details, critically concentrate on different parts of the environment and select between different options)	B13; F5	Prioritizing other interventions: [83]	Self-motivation and interest by patient to participate in PA: [64]
TDF domain 11: Environmental Context and Resources (This is an individual’s conditions that enables or prevents the development of expertise, social capabilities, and modifiable habits)	B1; F2	Lack of PA resources (e.g., education leaflets and materials): [31,63,70,73,82,88,89,90]	Assessable resources on PA promotional programmes (e.g., smart phone apps, assessment tools, etc.): [63,68,83,89]
		Inadequate staffing: [81]	Formal and central process for PA intervention: [71]
		Inaccessible PA supportive environment: [69,84]	Promotion of active treatment, home services and sporting activities: [64]
		Inadequate or lack of PA support services: [69,84]	
		Lack of specific PA guidelines: [65]	
		Limited counselling time: [31,52,53,63,65,67,71,73,76,78,80,82,86,88,89,90,92]	
		Lack of PA infrastructure and funding: [75]	
		Poor implementation and inconsistent support: [75]	
		Lack of PA facilities and funding: [64,75,84]	
		Paucity of PA specialist: [52,65,73,82]	
		Patient safeguarding procedures (i.e., for patient with mental health challenges): [81]	
		Long awaiting list to asses PA services: [69]	
		Transportation barrier: [77]	
		Lack of referral pathways for promotion: [52,67,86]	
TDF domain 12: Social influence (They are relational procedures that can influence the thinking and behavioral processes of a person)	B3; F4	Lack of support from practice or other colleagues: [82]	Encouraging informal communication strategies (e.g., building rapport, providing information, social support and understanding patient needs): [81]
		Lack of patient interest or motivation in PA: [31,64,67,68,69,73,80,85,89]	Opportunities for empathy and connection among HCPs and patients: [83]
		Patient preference for other intervention (e.g., drugs): [70]	
		Lack of cooperation among HCP’s: [69,83]	
TDF domain 13: Emotion (This is a complicated pattern of reaction, including practical, psychological and biological components which a person tries to use in the management of a crucial issue)	B9; F5	Feeling uncomfortable/inappropriate to speak to patient about PA: [82]	Opportunities for empathy and connection among HCPs and patients: [83]
		Perceived fear of liability and litigation: [52,71,86]	
TDF domain 14: Behavioural Regulation (This is anything intended for controlling or modifying a neutral event or measures)	B13; F5	Cultural restriction: [75]	Compulsory PA interventions to patients: [81]

**Table 4:** Quality assessment of the included studies.

	QATSDD Criteria																	
Author & Year	1	2	3	4	5	6	7	8	9	10	11	12	13	14	15	16	Total Score	Max Score (%)
Spellman et al. 2014 [31]	0	3	1	0	1	1	2	2	0	2	NA	2	1	NA	0	3	18/42	43
Barnes et al. 2019 [51]	0	3	3	2	3	3	0	2	0	3	NA	0	0	NA	0	2	21/42	50
Din et al. 2015 [52]	0	2	3	2	2	2	1	2	NA	NA	3	3	3	2	0	3	28/42	66.7
Haussmann et al. 2018 [53]	0	3	2	1	2	2	1	1	NA	NA	2	3	2	2	1	2	24/42	57.1
Cantwell et al. 2018 [63]	0	3	3	1	2	3	2	3	1	3	2	3	2	1	1	3	33/48	68.8
Eisele et al. 2020 [64]	0	3	3	2	3	3	2	2	NA	NA	2	3	3	2	3	3	34/42	81.0
Haussmann et al. 2018 [65]	0	3	2	1	3	3	2	3	0	2	NA	2	3	NA	2	3	29/42	69
Hurkmans et al. 2011 [66]	0	3	2	2	3	2	1	2	0	0	NA	2	3	NA	2	3	25/42	59.5
Jorgensen et al. 2012 [67]	0	2	2	0	2	2	1	1	2	2	NA	2	3	NA	2	3	24/42	57.1
Leemrijse et al. 2015 [68]	0	3	1	0	3	1	1	1	0	1	NA	1	0	NA	0	3	15/42	35.7
Mulligan et al. 2011 [69]	0	3	2	1	2	3	2	2	NA	NA	1	2	3	2	0	3	26/42	59
O’Brien et al. 2019 [70]	0	3	2	3	3	3	2	3	3	2	NA	3	2	NA	3	3	35/42	83.3
Sassen et al. 2011 [71]	3	3	2	1	3	3	1	2	0	2	NA	2	2	NA	1	2	26/42	62
Suija et al. 2010 [72]	0	0	3	1	2	2	1	3	1	2	NA	0	2	NA	0	3	20/42	47.6
Barrett et al. 2013 [73]	0	3	3	2	3	3	1	3	0	2	NA	2	0	NA	1	0	23/42	54.8
Burton et al. 2010 [74]	0	3	2	0	3	3	2	3	0	2	NA	2	3	NA	2	3	28/42	66.7
Frantz & Ngambare 2013 [75]	0	2	2	1	3	3	3	2	1	3	3	2	1	0	0	0	26/48	54.1
Freene et al. 2017 [76]	0	3	3	3	2	2	2	2	0	3	NA	3	3	NA	0	2	28/42	66.7
Patel et al. 2012 [77]	0	3	3	3	3	3	3	2	NA	NA	3	3	2	3	0	3	34/42	81.0
Patel et al. 2011 [78]	0	2	3	2	3	3	3	2	NA	NA	2	2	2	2	0	3	29/42	69
Shirley et al. 2010 [79]	0	3	1	1	3	2	2	2	0	2	NA	2	2	NA	0	3	23/42	54.8
Speake et al. 2019 [80]	0	3	2	3	3	3	3	2	NA	NA	2	3	3	3	2	3	35/42	83.3
Kinnafick et al. 2018 [81]	0	2	2	1	2	3	2	2	NA	NA	2	2	2	2	0	3	25/42	59.5
Litchfield et al. 2019 [82]	0	1	3	1	2	2	2	3	NA	NA	2	1	1	1	0	3	22/42	52.3
Lowe et al. 2018 [83]	3	2	2	3	3	2	2	3	NA	NA	3	3	3	2	0	3	34/42	81.0
Williams et al. 2018 [84]	2	3	3	3	3	3	3	2	NA	NA	3	3	2	3	0	2	35/42	83.3
Courtney-Long et al. 2017 [85]	0	3	2	0	2	3	1	2	0	1	NA	2	3	NA	0	2	21/42	50
Omura et al. 2018 [86]	0	3	2	1	2	3	2	3	0	3	NA	3	3	NA	0	3	28/42	66.7
Pearson et al. 2018 [87]	2	3	3	1	1	3	2	2	0	2	NA	3	2	NA	0	2	26/42	62
Pojednic et al. 2017 [88]	0	3	2	1	1	3	1	2	0	3	NA	2	0	NA	0	3	21/42	50
Tucker et al. 2017 [89]	0	2	1	1	2	2	2	2	0	2	NA	2	1	NA	0	2	19/42	45.2
Abaraogu et al. 2016 [90]	0	3	3	1	3	3	2	2	3	2	NA	2	1	NA	2	1	28/42	66.7
Patra et al. 2015 [91]	0	1	3	1	3	1	1	2	0	2	NA	2	2	NA	0	2	20/42	47.6
Freene et al. 2019 [92]	0	1	2	2	2	2	2	2	0	1	NA	3	3	NA	0	2	22/42	52.3

NA – Not Applicable Quality Assessment Tool for Studies with Diverse Designs (QATSDD) Criteria: (1) theoretical framework; (2) aims/objectives; (3) description of research setting; (4) sample size; (5) representative sample of target group, (6) procedure for data collection; (7) rationale for choice of data collection tool(s); (8) detailed recruitment data; (9) assessment of reliability and validity of measurement tool(s) (Quantitative only) (10) fit between research question and method of data collection (Quantitative only) (11) fit between research question and data collection method (Qualitative only) (12) fit between research question and method of analysis; (13) good justification for analytical method selected; (14) reliability of analytical process (Qualitative only); (15) evidence of user involvement in design (16) strengths and limitations. 0 = not at all; 1 = very slightly; 2 = moderately; 3 = complete; n/a = not applicable.

## Appendix A: Study Search Terms

“primary care” OR “primary healthcare” OR “Integrated health*” OR “primary healthcare p*” OR “patient car*” OR “healthcare p*” OR “general practi*” OR “family doctor*” OR doctor* Or gp* OR physician* OR surgeon* OR nurse* OR “physical therapist*” OR physio* OR “exercise physiologist*” OR “health p*” OR dietitian* OR “occupational therapist*” OR chiropractor* OR podiatrist* OR “allied health p*” AND

perception* OR know* OR inform* OR perspective* OR view* OR believe* OR opinion* OR idea* OR impression* OR proficiency OR “uptake and knowledge” OR behaviour AND

“physical activit*” OR exercise* OR sport* OR walk* OR run* OR “physical fitness” OR “exercise on referral” OR “physical activity on prescription” OR “exercise on prescription” OR “exercise is medicine” OR “green prescription” OR “exercise referral scheme” OR “physical activity promotion” OR “health promotion” AND inactiv* OR “chronic disease*” OR disease* OR sedentary OR “sedentary behaviour*” OR “lifestyle disease*” OR “life style, sedentary” OR “life style change*”

## Appendix B: Table Legend

Table 1: Study characteristics: This table provides a general information about all the included studies. Author: Study year and Reference: This column shows all the included studies, the first author and the study year of publication listed according to their order in the bibliography. Country of study: This column shows the countries where each of the studies originated from. Study design and study references: This column shows the study design employed in each of the studies and their reference number. Type of Healthcare Professional(s) (HCPs): This column portrays the healthcare professionals which perceptions were explored in the study. Participants (No.: Gender, Mean Age [yrs.]): This column portrays the total number of participants in the study, their sex and mean age.

Table 2: Participants’ perceptions about PA knowledge, confidence, importance, role assessment, recommendation and effectiveness: This table provides all the extracted views of healthcare professionals about all the key determinant factors impacting on effective PA promotion. Author: Study year and Reference: This column shows all the included studies, the first author and the study year of publication listed according to their order in the bibliography. Knowledge of PA: This column portrays HCPs’ views about their knowledge of PA promotion. Confidence in Promoting PA: This column portrays HCPs’ views about their confidence in promoting PA. Importance of PA and its Promotion: This column portrays HCPs’ views about the importance of promoting PA. Role in PA Promotion: This column portrays HCPs’ perceptions of their role in promoting PA.PA Assessment: This column portrays HCPs’ views about assessing for PA. How PA was promoted: This column portrays HCPs’ views about how they currently promote PA. Perceived Effectiveness of PA Promotion: This column portrays HCPs’ perceived effectiveness of PA and their effectiveness in promoting it. Table 3. Participants’ perceived barriers and facilitators to PA promotion: This table provides all the extracted perceptions of healthcare professionals about the barriers and facilitators to PA promotion based on the constructs of TDF domain.TDF Domains: This column portrays all the fourteen TDF domain factors and their individual definition.

Table 4. Quality assessment of the included studies: This table provides information on the quality assessment criteria used in this review.

Rank (B - Barrier and F-Facilitator): This is a ranking system used to grade all the TDF domain factors in hierarchy from 1 (highest) to 14 (least) based on how HCPs view each key TDF domain factors as either a barrier or a facilitator. Barrier constructs and Reference Number: This column portrays the views of HCPs about various barriers to PA promotion based on their categories into individual TDF domain constructs. Facilitator constructs and Reference Number: This column portrays the views of HCPs about various facilitators to PA promotion based on their categories into individual TDF domain constructs.

QATSDD criteria: This row shows a list of all the Quality Assessment Tool for Studies with Diverse Designs (QATSDD) item employed in this review. The QATSDD item were numbered from one to sixteen. The interpretation of the numbers includes: (1) theoretical framework; (2) aims/objectives; (3) description of research setting; (4) sample size; (5) representative sample of target group; (6) procedure for data collection; (7) rationale for choice of data collection tool(s); (8) detailed recruitment data; (9) assessment of reliability and validity of measurement tool(s) (Quantitative only); (10) fit between research question and method of data collection (Quantitative only); (11) fit between research question and data collection method (Qualitative only); (12) fit between research question and method of analysis; (13) good justification for analytical method selected; (14) reliability of analytical process (Qualitative only); (15) evidence of user involvement in design; (16) strengths and limitations. Author and Year: This column shows all the included studies and their year of publication listed according to their order in the references.
